# Update of a Genetic Risk Score Predictive of the Plasma Triglyceride Response to an Omega-3 Fatty Acid Supplementation in the FAS Study

**DOI:** 10.3390/nu15051156

**Published:** 2023-02-25

**Authors:** Ellie Gauthier, Juan de Toro-Martín, Bastien Vallée-Marcotte, Simone Lemieux, Iwona Rudkowska, Patrick Couture, Marie-Claude Vohl

**Affiliations:** 1 School of Nutrition, Université Laval, 2440 Hochelaga Blvd, Quebec City, QU G1V 0A6, Canada; 2 Centre Nutrition, Santé et Société (NUTRISS)-Institut Sur la Nutrition et les Aliments Fonctionnels (INAF), Université Laval, 2440 Hochelaga Blvd, Quebec City, QU G1V 0A6, Canada; 3 Endocrinology and Nephrology Unit, Département de Kinésiologie and CHU de Québec-Université Laval Research Center, Université Laval, Quebec City, QU G1V 0A6, Canada

**Keywords:** genetic risk score, omega-3 fatty acids, plasma triglycerides, interindividual variability

## Abstract

A genetic risk score (GRS) predictive of the plasma triglyceride (TG) response to an omega-3 fatty acid (*n*-3 FA) supplementation has been previously developed in the Fatty Acid Sensor (FAS) Study. Recently, novel single nucleotide polymorphisms (SNPs) interacting with a fish oil supplementation and associated with plasma lipid levels have been identified in the UK Biobank. The aim of this study was to verify whether the addition of SNPs identified in the UK Biobank to the GRS built in the FAS Study improves its capacity to predict the plasma TG response to an *n*-3 FA supplementation. SNPs interacting with fish oil supplementation in the modulation of plasma lipid levels in the UK Biobank and associated with plasma TG levels have been genotyped in participants of the FAS Study (*n* = 141). Participants have been supplemented with 5 g fish oil/day for six weeks. Plasma TG concentrations were measured before and after the supplementation. Based on the initial GRS of 31 SNPs (GRS31), we computed three new GRSs by adding new SNPs identified in the UK Biobank: GRS32 (rs55707100), GRS38 (seven new SNPs specifically associated with plasma TG levels), and GRS46 (all 15 new SNPs associated with plasma lipid levels). The initial GRS31 explained 50.1% of the variance in plasma TG levels during the intervention, whereas GRS32, GRS38, and GRS46 explained 49.1%, 45.9%, and 45%, respectively. A significant impact on the probability of being classified as a responder or a nonresponder was found for each of the GRSs analyzed, but none of them outperformed the predictive capacity of GRS31 in any of the metrics analyzed, i.e., accuracy, area under the response operating curve (AUC-ROC), sensitivity, specificity and McFadden’s pseudo R^2^. The addition of SNPs identified in the UK Biobank to the initial GRS31 did not significantly improve its capacity to predict the plasma TG response to an *n*-3 FA supplementation. Thus, GRS31 still remains the most precise tool so far by which to discriminate the individual responsiveness to *n*-3 FAs. Further studies are needed in the field to increase our knowledge of factors underlying the heterogeneity observed in the metabolic response to an *n*-3 FA supplementation.

## 1. Introduction

Numerous studies suggest that the consumption of marine omega-3 fatty acids (*n*-3 FAs), i.e., eicosapentaenoic acid (EPA) and docosahexaenoic acid (DHA) may reduce the risk of cardiovascular events, by decreasing plasma triglyceride (TG) levels [1,2]. However, there is a large interindividual variability in the metabolic response to *n*-3 FAs [3]. In the Fatty Acid Sensor (FAS) Study conducted by our group, it was reported that 29% of the participants did not lower their plasma TG levels after a six-week supplementation of 5 g of fish oil providing 1.9–2.2 g EPA and 1.1 g DHA daily. It is well demonstrated in the literature that this interindividual variability is partly due to genetic factors [4].

A genome-wide association study (GWAS) revealed genetic markers associated with the plasma TG response in participants of the FAS Study. Significant GWAS signals were found in 6 loci: *IQCJ-SCHIP1*, *NXPH1*, *PHF17*, *MYB*, *NELL1*, and *SLIT2* [4]. After fine mapping, 31 single nucleotide polymorphisms (SNPs) were used to calculate a genetic risk score (GRS), which explained 49.7% of the variance in the plasma TG levels during the supplementation protocol [5]. According to this GRS31, carriers of more risk alleles are more likely to be nonresponders and not to lower their plasma TG levels following the *n*-3 FA supplementation [5]. 

Similarly, a recent GWAS on 73,962 individuals of the UK Biobank identified new loci interacting with fish oil supplementation to modulate plasma lipid levels [6]. More precisely, they identified gene–diet interactions with fish oil supplementation and influencing either plasma TG, low-density cholesterol (LDL-C), high-density cholesterol (HDL-C), or total cholesterol (TC) levels by using longitudinal data. These SNPs were all individually located in different genes, with those associated with plasma TG levels being in *GJB6*, *BAZ1B*, *LOC107986921*, *MAP1A*, and *HAPLN4* genes. 

These newly identified SNPs were of particular interest because of significant gene–diet interactions with *n*-3 FA supplementation, and they were located in different genes than those previously identified in the FAS Study. We then hypothesized that their addition to the previously developed GRS31 may improve its capacity to discriminate between different phenotypes of plasma TG response to an *n*-3 FA supplementation. The objective of the present study was then to test whether the addition of SNPs identified in the UK Biobank may improve the predictive capacity of the GRS31 built in the FAS Study, which will, ultimately, help predict an individual’s plasma TG response to an *n*-3 FA supplementation.

## 2. Materials and Methods

### 2.1. FAS Study Population

A total of 254 healthy subjects from the Quebec City metropolitan area were recruited into the FAS Study from 2009 to 2011. Participants had a body mass index (BMI) between 25 kg/m^2^ and 40 kg/m^2^, were aged between 18 and 50 years old, were nonsmokers, and were free from metabolic or thyroid disorder requiring a pharmacological treatment. Subjects were excluded if they had taken *n*-3 FA supplements for a minimum of six months prior to the intervention. A total of 210 participants completed the intervention protocol. The experimental protocol was approved by the ethics committees of Laval University Hospital Research Center and Laval University. This nontrial was registered at www.clinicaltrials.gov as NCT01343342. Plasma TG levels of two participants were unavailable for further analyses, which excluded them from the final sample. The 208 remaining subjects were subsequently separated into two subgroups: responders and nonresponders. Responders were defined as subjects whose plasma TG levels decreased after the *n*-3 FA supplementation (change in TG levels ≤ 0.01 mM) whereas nonresponders had increased or stable plasma TG levels (change in TG of ≥ 0.00 mM), as previously described [4]. From these groups, GWAS was done on a total of 141 participants showing the most extreme plasma TG response to an *n*-3 FA supplementation, of which 81 were responders (showing at least a 10% decrease) and 60 were nonresponders. These 141 participants with available GWAS data were included in the present study.

### 2.2. Study Design

Study design and diets have been previously reported [7,8]. Briefly, all participants from the FAS Study followed a run-in period of two weeks, during which they were given dietary instructions by a trained registered dietitian in order to achieve dietary recommendations from Canada’s Food Guide [9]. The aims of these instructions were to maintain a stable body weight and control participants’ *n*-3 FA intake throughout the intervention protocol. After the run-in period, subjects were asked to take five capsules per day of fish oil for six weeks. Capsules provided a daily intake of 1.9–2.2 g EPA and 1.1 g DHA. Blood samples were collected before and after the supplementation. Prior to the collection, participants had to fast for 12 h and abstain from alcohol consumption for 48 h. Lipid concentrations were measured by enzymatic assays as previously described [4]. 

### 2.3. SNP Genotyping

All 14 SNPs identified in the UK Biobank were genotyped in the 141 participants of the FAS Study [5,6]. Genomic data from the blood samples were first extracted by using the GenElute Gel Extraction Kit (Sigma-Aldrich Co., St. Louis, MO, USA). Genotyping was performed by Agena MassARRAY platform with iPLEX gold chemistry at the Centre d’expertise et de services Génome Québec [10]. Three extra SNPs (rs11983997, rs80189144 and rs147166404) in linkage disequilibrium (r^2^ > 0.5) with rare SNPs (MAF < 0.01) were also genotyped by using TaqMan technology at the Institute of Nutrition and Functional Foods at Université Laval, which formed an initial sample of 17 SNPs. Genotyping failed for one participant, leading a final cohort of 140 subjects. 

### 2.4. GRS Construction

PLINK software [11] was used to compare SNP allele frequency between responders and nonresponders, calculate the SNPs’ minor allele frequency (MAF) and conduct a Hardy–Weinberg equilibrium (HWE) test for each SNP. HWE’s significance threshold was set at *p* = 0.05. These analyses revealed two monomorphic SNPs (rs147166404 and rs530804537) in the FAS Study, i.e., none of the participants carried the rare allele. Both SNPs were excluded from further analysis, yielding a final total of 15 SNPs, of which seven were specifically associated with plasma TG levels in the UK Biobank. The odds ratios (OR) of the proportion of nonresponders over responders carrying the minor allele with a 95% confidence interval (95% CI) were used to define a risk score for each SNP. A score of 1 was attributed to SNPs associated with an increase of plasma lipid levels after the *n*-3 FA supplementation (OR < 1), whereas a score of −1 was attributed to SNPs associated with a decrease of plasma lipid levels (OR > 1). SNPs with MAF = 0 in the responder group were excluded from the construction of the GRS. In total, three new GRSs were calculated for each participant by summing up the score of risk alleles of the 31 SNPs (GRS31) from our initial GRS with: 1- GRS32 (addition of rs55707100); 2- GRS38 (addition of seven SNPs specifically associated with plasma TG levels in the UK Biobank); 3- and GRS46 (addition of all 15 SNPs from the UK Biobank). 

### 2.5. Statistical Analyses

Statistical analyses were performed on SAS software version 9.4. Differences between responders and nonresponders before and after the *n*-3 FA supplementation were assessed with a two-tailed unpaired *t*-test. Independent associations between SNPs and changes in lipid traits in the FAS Study were assessed with linear mixed models (MIXED procedure in SAS) adjusted for age, sex, and BMI as fixed effects, and individuals as random effects to account for within-subject variability. Statistical significance was set at *p* < 0.05. The GLM procedure in SAS was used to generate a general linear model adjusted for age, sex, and BMI, which evaluated the contribution of the GRSs to plasma TG levels after the *n*-3 FA supplementation. 

### 2.6. Evaluation of the Predictive Performance

The predictive performance of the different GRSs was assessed by calculating the area under the receiver operating characteristic curve (AUC-ROC) by using the logistic procedure. Individual prediction models were created for each of the clinical predictors (age, sex, BMI, and baseline plasma TG levels), as well as for each of the GRSs analyzed. Final prediction models were created by the sequential addition of clinical predictors and the given GRS. By using the R software (v4.2.1), the population study was then randomly split into training and testing datasets at 50%, with a balanced ratio of responders and nonresponders in each dataset, where models were trained and tested. The R caret package was used to perform a tenfold cross-validation in the training dataset. The cross-validated model obtained in the training dataset was further assessed in the testing dataset. The AUC-ROC and the accuracy were used to evaluate the predictive performance of each GRS in training and testing datasets. With the accuracy standing for the proportion of true responders and nonresponders out of the total number of subjects. The McFadden’s pseudo R^2^ was also used to determine the proportion of response variation accounted for by each GRS, which is an estimation of their predictive power. 

## 3. Results

### 3.1. Characteristics of Participants

Characteristics of participants in the FAS Study have already been reported [4]. Both responders and nonresponders were overweight or presented obesity at baseline, with a mean BMI of 28.9 ± 3.6 and 27.9 ± 3.9 kg/m^2^, respectively. More precisely, among responders, 65.4% were overweight and 34.6% presented obesity, whereas among nonresponders, 79.7% were overweight and 20.3% presented obesity. Responders had higher plasma TG levels at baseline than nonresponders (*p* < 0.001). After the *n*-3 FA supplementation, responders significantly decreased their plasma TG levels by 0.50 ± 0.36 mmol/L (*p* < 0.001), whereas nonresponders increased them by 0.18 ± 0.17 mmol/L (*p* < 0.001). Changes in BMI (*p* < 0.01), plasma TG (*p* < 0.0001), HDL-C (*p* = 0.0002), and TC (*p* = 0.01) levels after the *n*-3 FA supplementation were significantly different between the two subgroups, whereas changes in LDL-C levels were similar (*p* = 0.6).

### 3.2. Association between SNPs and Plasma Lipid Traits

Main characteristics of the 15 SNPs interacting with fish oil supplementation and associated with plasma lipid levels in the UK Biobank cohort and genotyped in participants of the FAS Study are presented in Table 1. All SNPs were in HWE. Allele frequencies of each SNP in both responders and nonresponders are shown in Table 2. Associations of all 15 SNPs with plasma TG, LDL-C, HDL-C, and TC levels in individuals from the FAS Study are presented in Table 3. None of the SNPs interacted with *n*-3 FA to modulate plasma TG levels in the FAS Study. 

### 3.3. Evaluation of Genetic Risk Scores

**Figure 1:**
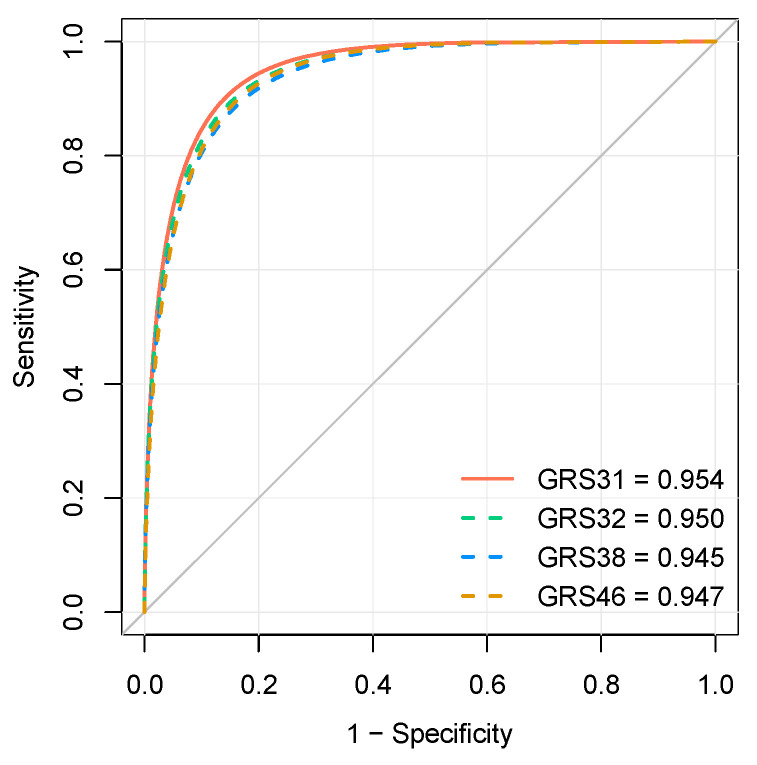
Prediction performance of the genetic risk scores. From left to right and from top to bottom are shown the receiver operating characteristic (ROC) curves of the final prediction models, including age, sex, body mass index, baseline triglycerides, and genetic risk score, for GRS31, GRS32, GRS38, and GRS46 in the FAS cohort.

**Figure 2:**
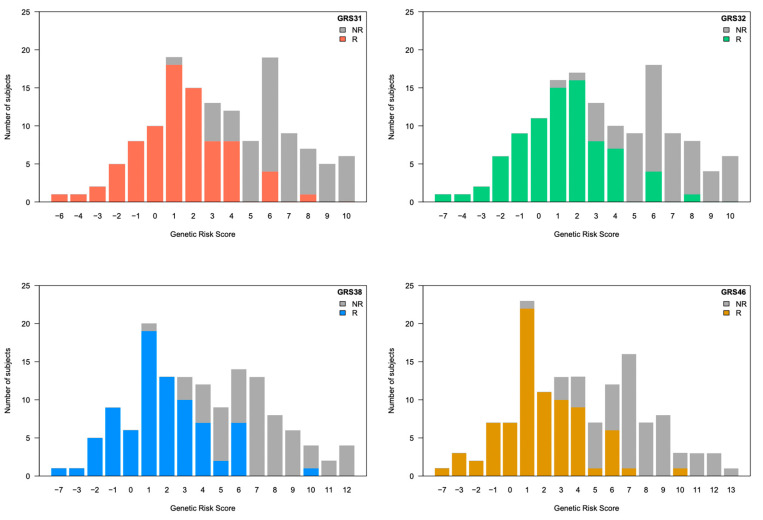
Distribution of genetic risk scores in the study sample. From left to right and from top to bottom are shown the stacked histograms for the distribution of GRS31, GRS32, GRS38, and GRS46 in the study population. GRS, genetic risk score. R, responders; NR, nonresponders.

**Figure 3:**
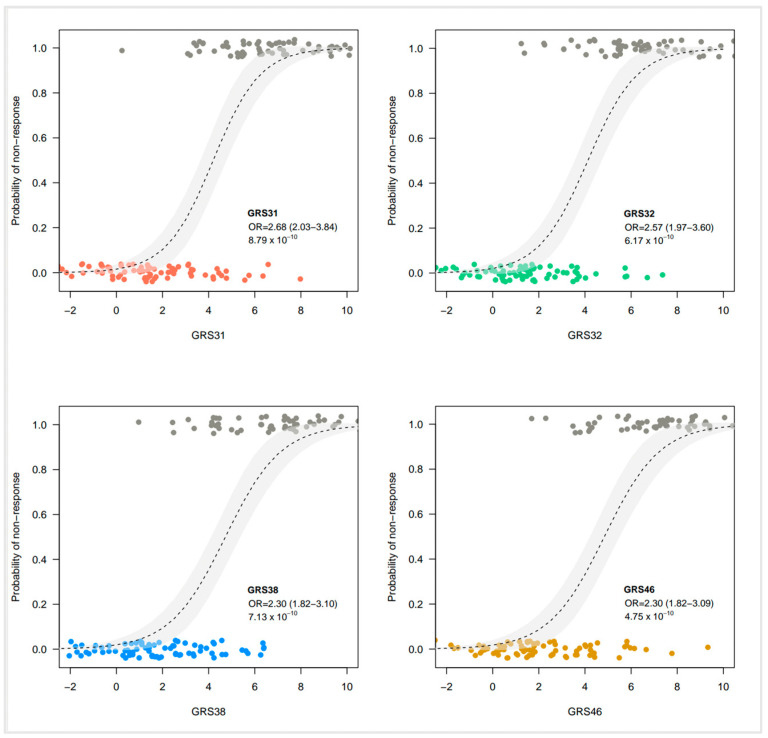
Impact of genetic risk scores on *n*-3 fatty acids responsiveness. From left to right and from top to bottom are shown the logistic curves of the final models for GRS31, GRS32, GRS38, and GRS46 The association between genetic risk scores and the probability of being a nonresponder is shown. Gray shaded areas represent 95% confidence intervals. OR, odds ratio.

We first tested the effect of adding one SNP of interest (rs55707100) to GRS31. The SNP rs55707100, included in GRS32 was of particular interest because it is a missense variant with a MAF of 4.6% in the FAS Study and was associated with plasma TG levels in the UK Biobank. Originally, GRS31 explained 49.7% of the variance in the change of plasma TG levels in the sample of 141 participants. Herein, after the exclusion of one participant due to genotyping failure, GRS31 accounted for 50.1% of the variance in plasma TG levels following the *n*-3 FA supplementation. The addition of rs55707100 in GRS32 did not show an incremental effect on the explained variance of GRS31 and slightly reduced it to 49.1%. Although the predictive capacity of the GRS31, measured as the AUC-ROC, was 0.954, the addition of rs55707100 in GRS32 set it to 0.950. The inclusion of additional SNPs did not lead to a further rise in the percentage of explained variance with GRS38 (45.9%) or GRS46 (45.0%) (Figure 1). Similarly, an incremental effect on the predictive capacity was not observed with GRS38 (AUC-ROC = 0.945) or GRS46 (AUC-ROC = 0.947), as compared to GRS31 (Figure 1). Individual prediction models for each of the clinical predictors, as well as for each GRS are shown in Figure S1. The increasing risk of nonresponse to *n*-3 FA supplementation, illustrated here by increasing GRS values, indicates that a subject carries a greater number of risk alleles, as shown in Figure 2. A statistically significant impact on the probability of being classified as a responder or a nonresponder was found for each of the GRS analyzed (Figure 3). Weighted versions of GRS32, GRS38, and GRS46 were also developed by using interaction effect sizes. Indeed, in the UK Biobank study, large effect sizes (greater than 0.8) were observed for most of SNPs showing significant interaction effects [6]. However, the weighted GRSs did not improve the prediction capacity of unweighted versions tested in the FAS Study, with explained variances of 35.3%, 36.4%, and 35.5% for weighted versions of GRS32, GRS38, and GRS46, respectively. 

### 3.4. Comparison of Genetic Risk Scores

The evaluation of the performance of each GRS to predict the plasma TG response was assessed in both training and testing datasets after applying tenfold cross-validation. GRS31 showed an accuracy of 0.93 [95% CI 0.84–0.98] in the training dataset and 0.77 [95% CI 0.66–0.86] in the testing dataset (Table 4; Figure S2). GRS31 also showed a high AUC-ROC in both training (AUC-ROC = 0.97 [95% CI 0.93–0.99]) and testing datasets (AUC-ROC = 0.87 [95% CI 0.79–0.95]) (Table 4; Figure S3). More precisely, the sensitivity i.e., the proportion of responders correctly identified as such, decreased from 0.93 in the training dataset to 0.73 in the testing dataset. Similarly, the specificity, i.e., the proportion of nonresponders correctly identified as such, decreased from 0.92 during training to 0.80 in the testing dataset. As expected, the proportion of explained variance in the plasma TG response decreased from 0.66 in the training dataset to 0.48 in the testing dataset. None of the GRSs with newly added SNPs outperformed the predictive capacity of GRS31 in any of the metrics analyzed, i.e., accuracy, area under the response-operating curve (AUC-ROC), sensitivity, specificity, and McFadden’s pseudo R^2^ (Table 4).

### 3.5. Genetic Risk Score Threshold Selection

The GRSs built in this study are aimed at aiding in clinical decision-making regarding treatment choice. Thus, a tradeoff between specificity and sensitivity has to be taken into account when considering the GRS threshold selection to increase *n*-3 FA treatment efficacy. Moreover, in order to maximize the potential number of patients to be treated, the correct identification of responders to the *n*-3 FA supplementation (sensitivity) need to be favored over the accurate classification of nonresponders (specificity).

**Figure 4:**
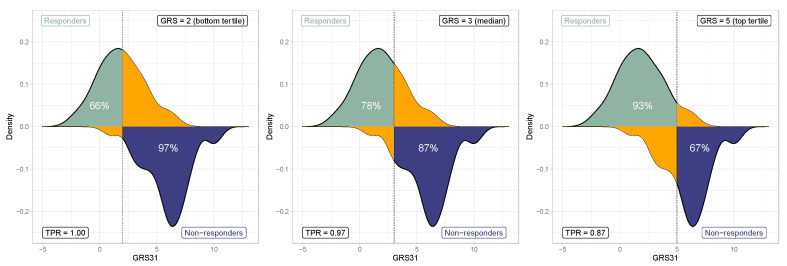
Genetic risk score threshold selection. From left to right is shown the distribution of responders and nonresponders at the bottom tertile, median, and top tertile of GRS31. Percentages within density plots represent the actual proportion of responders and nonresponders. Orange areas represent responders and nonresponders outside the selected threshold. TPR, true positive rate (sensitivity).

**Figure 5:**
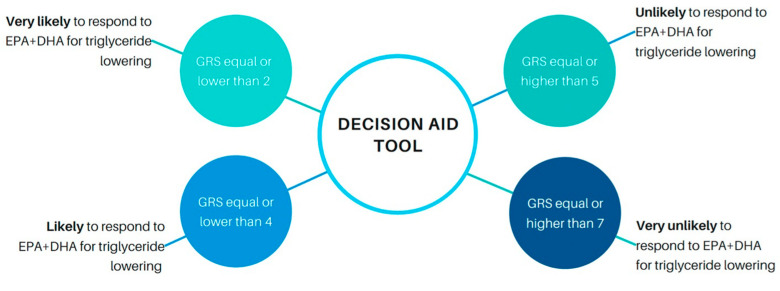
Decision aid tool prototype for the identification of subjects more likely to benefit from the TG lowering effects of *n*-3 FA. GRS, genetic risk score; EPA, eicosapentaenoic acid; DPA, docosahexaenoic acid.

By using the median score value of GRS31 (GRS31 = 3), 78% of actual responders would have an equal or lower score, whereas 97% of nonresponders would have equal or higher scores (Figure 4). This means that, by using the testing prediction outcomes of the GRS31 model, this cutoff would lead to a sensitivity of 97%. At bottom and top tertiles of GRS31, the predicted probability of being identified as responder (GRS31 < 2) or nonresponder (GRS31 > 5) would be higher than 80% for both, and the accuracy of the model would increase from 77% to 89%. According to this, setting the GRS cutoff to 2 would increase the sensitivity of the model (the probability for an actual responder of being correctly classified as responder) to 100%. However, the percentage of actual responders having a GRS equal or lower than 2 would be reduced to only 66% (Figure 4), which means that 44% of potential responders would not be considered for *n*-3 FA treatment. Given that the goal of a decision aid for *n*-3 FA plasma lipid responsiveness would be to maximize the number of subjects to be treated, increasing the GRS cutoff would be preferred. Setting the threshold to 5, the top tertile of GRS31, would lead to a decrease in sensitivity to 87%, but the tool would be able to capture the majority of potential responders (concretely 93% of them (Figure 4)). We may then consider a GRS31 equal or less than 5 as an optimal cutoff to identify potential responders to a *n*-3 FA supplementation in terms of plasma TG response. At this threshold, only 1 out of 10 actual nonresponders would be identified as a responder, while only 1 out of 8 actual responders would not be accurately identified as such. Based on these findings, we illustrated a decision aid tool prototype to identify individuals that would be more likely to benefit from the plasma TG lowering effect of *n*-3 FAs (Figure 5). 

## 4. Discussion

The aim of the present study was to test whether the addition of new SNPs identified in a recent UK Biobank study [6] to the GRS built in the FAS Study improved its capacity to predict the plasma TG response to an *n*-3 FA supplementation. A total of three GRSs was built from the addition of these new SNPs. On the whole, none of these GRSs managed to marginally increase the prediction capacity of the original GRS31. 

Moreover, it is important to point out that after quality control, a total of 7,954,107 autosomal variants from Affymetrix UK Biobank Axiom and Affymetrix UK BiLEVE Axiom arrays were included in the analyses of UK Biobank. From them, only 5 out of the 31 SNPs of our GRS31 were present in the arrays used in the UK Biobank. These SNPs are rs12702829 (*NXPH1*), rs72974149 (*MYB*), rs114348423 (*PHF17*), rs117114492, and rs78786240 (*NELL1*). None of these SNPs significantly interacted with fish oil consumption in the modulation of plasma lipid levels in the UK Biobank.

The most plausible explanation to these results is the divergence between study designs. Francis et al. conducted a GWAS on participants of the UK Biobank being a longitudinal study [6], whereas the FAS Study was a clinical intervention trial. More precisely, UK Biobank participants’ supplementation status was assessed with a medical questionnaire, making it impossible to ascertain the frequency at which fish oil supplements were taken as well as the quantity of *n*-3 FA consumed daily. Participants of the FAS Study, however, followed a controlled protocol in which frequency and adherence to the *n*-3 FA supplementation where monitored. In addition, it is highly plausible that the daily consumption of EPA and DHA in the UK Biobank study was much lower than the daily intake of 1.9–2.2 g EPA and 1.1 g DHA in the FAS Study, given that many *n*-3 FA capsules sold on the market do not contain as much EPA and DHA. These differences could therefore explain why SNPs identified in the UK Biobank study did not improve the prediction capacity of GRS31. Finally, even though Francis et al.’s study design has a good power to detect gene–diet interactions in a statistical model, there is no possibility to directly assess or predict the plasma TG response to an *n*-3 FA supplementation. These SNPs interact with *n*-3 FA supplementation and are associated with plasma TG levels but not necessarily with the plasma TG responsiveness to an *n*-3 FA supplementation as tested in the FAS Study. 

Another factor that could have contributed to the absence of stronger results is the large size difference between the two study samples. The novel genetic variants interacting with an *n*-3 FA supplementation to modulate plasma TG levels in the study of Francis et al. were identified after performing analyses on 73,962 individuals [6]. The genotyping of these new variants was conducted on 140 participants in the FAS Study. Correspondingly, most genotyped SNPs showed a weak MAF, which substantially decreased our ability to construct more qualified GRSs. Moreover, ancestry background may, at least partly, explain why genetic variants identified in the UK Biobank did not improve the prediction capacity of the GRS built in the FAS Study. UK Biobank subjects included in Francis et al.’s study are British Caucasian [6], whereas FAS participants are French Canadians of European descent, which form a more homogenous founder population [12]. Given the ethnical differences between these two studies and the founder effect in the French Canadian population, it is plausible that the potential SNPs that modulate the association between *n*-3 FA supplements and the plasma TG response have different frequencies in each study. This hypothesis could explain why the SNPs identified in the UK Biobank study did not improve our GRS31 from the FAS Study. 

We initially built GRS32 by including the SNP rs55707100, a missense variant located within the *MAP1A* gene. The inclusion of this SNP was based on its significant association with plasma TG levels in the UK Biobank, as well as on its relatively high MAF of 4.6% compared to most other SNPs, which would increase our ability to obtain meaningful results in the much shorter FAS Study. Missense variants are alterations of the nucleotide coding sequence that result in a substitution of the initial amino acid. They are of particular interest because the amino acid substitution can alter the function of the protein and lead to disease development, or in this case, interact with the plasma TG response following an *n*-3 FA supplementation [13]. The SNP rs55707100 was also associated with plasma TG levels in past studies conducted on large samples of individuals [14,15]. In view of these previous findings, we hypothesized that the addition of rs55707100 to GRS31 would improve the prediction capacity of the initial GRS31 more than what was actually observed. Our results then make it difficult to ascertain the degree of association of rs55707100 with plasma TG responsiveness to an *n*-3 FA supplementation.

As for GRS38, only SNPs interacting with plasma TG levels in Francis et al.’s study were added to the initial GRS31. In GRS46, we included all 15 new SNPs in order to verify whether SNPs associated with other lipid parameters and interacting with *n*-3 FA supplementation in the UK Biobank cohort also modulate plasma TG responsiveness in the FAS Study. This hypothesis comes from the fact that plasma HDL-C and TC levels were also diminished alongside plasma TG levels in the FAS Study. Moreover, most of the SNPs interacting with *n*-3 FA supplementation to modulate plasma TG levels in the UK Biobank study are located in genes previously associated with other lipid traits, which made these SNPs even more attractive for our study. *GBJ6* encodes one of the connexin proteins in cell’s gap junction in multiple tissues [16]. *BAZ1B* encodes a protein involved in chromatin-dependent regulation of transcription [17], and it has been associated with plasma lipid profiles in Chinese patients with type 2 diabetes [18]. *MAP1A* is mostly expressed in the brain and is involved in microtubule assembly, an important step in neurogenesis [19], and it has also been linked to lipid metabolism [13]. As for *HAPLN4*, it has been predicted to be involved in the central nervous system, skeletal and hyaluronic acid binding activity [20], and it has been also identified as a common variant associated with plasma levels of different lipid species and with coronary artery diseases in a GWAS performed by Cadby et al. on individuals from the Busselton Health Study and the UK Biobank [21]. The *LPL* gene encodes for the lipoprotein lipase [22], an enzyme mostly expressed in the heart, skeletal muscle, and brown and white adipose tissue, which plays a role in fatty acid oxidation and storage. Mutations located in the *LPL* gene can cause hyperlipoproteinemia and lipid metabolism disorders [23,24]. As for *MLXIPL*, it encodes a transcription factor that activates the carbohydrate response element motifs in the plasma TG synthesis genes [25]. Moreover, a recent study from the Global Lipids Genetics Consortium provided a list of 32 loci modulating plasma TG levels, which included *MLXIPL* [26]. Similarly, SNPs in *SLC12A3*, a gene that encodes for a sodium-chloride cotransporter important for electrolyte homeostasis [27], were associated with lipid profiles in Mongolian and Chinese populations [28,29,30]. Finally, the gene *ABCA6* encodes for the ATP binding-cassette transporter [31] and has been associated with cholesterol levels in a GWAS performed in nine Dutch biobanks [32]. *LOC107985305* also interacted with LDL-C levels in multiomics analysis performed by Michelle et al. [33].

These previous findings led us to test whether the SNPs identified in Francis et al.’s study were associated with plasma TG responsiveness to an *n*-3 FA supplementation. With most of these genes being associated with plasma lipid levels in past studies, it was relatively unexpected not to find stronger associations with plasma TG levels in the present study. Moreover, neither of these two approaches, either the seven SNPs specifically interacting with plasma TG levels (GRS38), or all the 15 SNPs identified in the UK Biobank (GRS46) improved the prediction capacity of GRS31. Thus, the initial GRS31 remains the best tool so far by which to discriminate responders from nonresponders to an *n*-3 FA supplementation [5]. As already mentioned, the fact that the UK Biobank results were based on a longitudinal study and the FAS Study was a nutritional intervention and then seek for genes associated with plasma TG responsiveness seems to be on the basis of the lack of expected results. Nevertheless, it is worth highlighting that GRS construction is a dynamic and continuous task that requires a continuous feeding with novel and promising variants.

The evaluation used to compare the predictive capacity of the different GRSs was appropriately assessed herein by using cross-validation and by splitting the entire cohort into training and testing datasets. However, the sample size of the cohort on which the study relies, as well as the modest level of association between the plasma TG response and newly added SNPs, may constitute limitations. Moreover, these GRSs are based on French Canadians and European descent populations, which potentially misestimates the risk prediction if applied to other populations, especially of non-European descent. 

The ultimate goal of a GRS for the plasma TG response is the development of a precision tool for the identification of subjects more likely to benefit from the plasma TG lowering effects of *n*-3 FA. Thus, a genetic-informed estimation of *n*-3 FA responsiveness was drafted herein as a previous step in the development of a decision aid prototype, illustrated in Figure 5. The rationale behind GRS threshold selection is explained from the fact that the incorrect identification of a nonresponder to *n*-3 FA as a responder would not have severe consequences for the patient. In contrast, the incorrect identification of an actual responder as a nonresponder would prevent potential patients to be treated. Thus, giving *n*-3 FAs to all the potential responders, at the expense of decreasing sensitivity, was preferred here. As previously discussed, this type of genetic-informed decision tools is increasingly being used to guide clinical practice for healthcare professionals [34]. However, further research including larger, heterogeneous and comprehensive cohorts, are still needed to develop accurate decision aids related to *n*-3 FA responsiveness regarding plasma TGs, in order to support its potential widespread use.

## 5. Conclusions

In conclusion, the addition of the novel SNPs identified in UK Biobank to the GRS initially built and refined in the FAS Study did not significantly improve its capacity to predict the plasma TG response to an *n*-3 FA supplementation. Therefore, the initial GRS31 remains the most precise tool so far to discriminate responders from nonresponders in the plasma TG responsiveness to an *n*-3 FA supplementation. 

## Figures and Tables

**Table 1:** SNPs characteristics and distribution in the FAS Study.

SNP	CHR	BP	Nearest Gene (Location)	Major/Minor Allele	MAF (%)	HWE P	Phenotype
rs115675705	6	34,094,919	*GRM4* (intron)	T/C	1.4	1	HDL
rs117788606	7	72,921,771	*BAZ1B* (intron)	A/G	0.7	1	TG
rs11983997	7	72,939,244	*BAZ1B* (upstream)	C/G	20.0	0.2	TG
rs80189144	7	72,939,939	*BAZ1B* (upstream)	T/C	13.2	0.1	TG
rs799157	7	73,020,301	*MLXIPL* (synonymous)	C/T	3.9	1	LDL
rs117860853	8	19,722,204	*LPL* (upstream)	G/A	1.8	1	HDL
rs142084074	8	19,768,150	*LOC107986921* (intron)	G/A	0.7	1	TG
rs144018203	11	116,916,060	*SIK3* (intron)	G/C	0.7	1	HDL.TG
rs112803755	13	20,790,451	*GJB6* (downstream)	A/G	1.4	1	TG
rs55707100	15	43,820,717	*MAP1A* (missense)	C/T	4.6	1	TG
rs148931404	16	56,914,455	*SLC12A3* (intron)	C/T	1.1	1	HDL
rs147438979	17	42,061,277	*PYY* (intron)	G/C	1.8	1	HDL
rs77542162	17	67,081,278	*ABCA6* (missense)	T/C	0.4	1	LDL.TC
rs141844019	19	19,365,178	*HAPLN4* (downstream)	G/A	1.1	1	TG
rs112952132	19	45,198,060	*LOC107985305* (intron)	G/A	0.7	1	LDL

CHR, chromosome; BP, base pair; MAF, minor allele frequency; HWE P, Hardy–Weinberg equilibrium *p*-value; LDL, low-density cholesterol; HDL, high-density cholesterol; TC, total cholesterol. Genes: *GRM4*, glutamate metabotropic receptor 4; *BAZ1B*, bromodomain adjacent to zinc finger domain 1B; *MLXIPL*, MLX interacting protein like; *LPL*, lipoprotein lipase; *SIK3*, SIK family kinase 3; *GJB6*, gap junction protein beta-6; *MAP1A*, microtubule associated protein 1A; *SLC12A3*, solute carrier family 12 member 3; *PYY*, peptide YY; *ABCA6*, ATP binding cassette subfamily A member 6; *HAPLN4*, hyaluronan and proteoglycan link protein 4; *LOC107986921* and *LOC107985305* are uncharacterized. SNPs underlined are substitutes for rare SNPs (MAF < 0.01).

**Table 2:** SNPs allele frequencies in the FAS Study and genetic risk score values.

	MAF (%)				
SNP	R	NR	OR	(95% CI)	GRS
rs115675705	0.01	0.02	0.73	(0.10–5.22)	1
rs117788606	0.01	0.01	0.74	(0.05–11.9)	1
rs11983997	0.18	0.23	0.73	(0.41–1.32)	1
rs80189144	0.12	0.14	0.84	(0.42–1.68)	1
rs799157	0.03	0.05	0.59	(0.18–2.00)	1
rs117860853	0.01	0.03	0.48	(0.08–2.91)	1
rs142084074	0.01	0.01	0.74	(0.05–11.9)	1
rs144018203	0.01	0.01	0.73	(0.04–11.7)	1
rs112803755	0.01	0.02	0.74	(0.10–5.31)	1
rs55707100	0.06	0.03	1.71	(0.51–5.68)	−1
rs148931404	0.01	0.01	1.46	(0.13–16.3)	−1
rs147438979	0.02	0.01	2.96	(0.33–26.9)	−1
rs77542162	0.00	0.01	-	-	-
rs141844019	0.01	0.01	1.49	(0.13–16.6)	−1
rs112952132	0.01	0.01	0.73	(0.04–11.7)	1

MAF, minor allele frequency; R, responders; NR, nonresponders; GRS, genetic risk score; SNPs underlined are substitutes for rare SNPs (MAF < 0.01). ORs, odds ratio of the proportion of nonresponders over responders carrying the minor allele with a 95% confidence interval (95% CI). A score of 1 is attributed to SNPs associated with an increase of plasma lipid levels after the *n*-3 FA supplementation (OR < 1), whereas a score of −1 is attributed to SNPs associated with a decrease (OR > 1).

**Table 3:** SNPs associations in the FAS Study with plasma lipid traits.

	*p*-Value (Treatment-by-Visit Interaction Term)			
SNP	TG	LDL-C	HDL-C	TC
rs115675705	0.96	0.21	0.04 *	0.11
rs11983997	0.43	0.25	0.02 *	0.02 *
rs80189144	0.90	0.51	0.01 *	0.06
rs799157	0.29	0.52	0.37	0.43
rs117860853	0.35	0.81	0.73	0.86
rs144018203	0.58	0.33	0.14	0.10
rs112803755	0.98	0.19	0.48	0.11
rs55707100	0.67	0.60	0.65	0.66
rs148931404	0.53	0.14	0.79	0.17
rs147438979	0.86	0.43	0.46	0.73
rs77542162	0.06	0.34	0.93	0.19
rs112952132	0.72	0.33	0.71	0.30
rs117788606	0.70	0.33	0.92	0.41
rs141844019	0.87	0.09	0.001 *	0.02 *
rs142084074	0.84	0.11	0.001 *	0.02 *

SNPs underlined are substitutes for rare SNPs (MAF < 0.01). TG, triglycerides. LDL-C, low-density cholesterol; HDL-C, high-density cholesterol; TC, total cholesterol. Asterisks stand for statistical significance at *p* ≤ 0.05 for the treatment-by-visit interaction term, calculated by means of a linear mixed model adjusted for age, sex and BMI.

**Table 4:** Predictive performance of genetic risk scores in training and testing datasets.

	GRS31		GRS32		GRS38		GRS46	
	Train	Test	Train	Test	Train	Test	Train	Test
Accuracy	0.93	0.77	0.93	0.77	0.91	0.76	0.90	0.77
AUC-ROC	0.97	0.87	0.97	0.86	0.97	0.84	0.97	0.85
Sensitivity	0.93	0.73	0.93	0.73	0.90	0.70	0.86	0.73
Specificity	0.92	0.80	0.92	0.80	0.92	0.80	0.92	0.80
McFadden	0.66	0.48	0.64	0.48	0.61	0.45	0.61	0.46

GRS, genetic risk score; AUC-ROC, area under the response-operating curve.

## Data Availability

The data presented in this study are available on request from the corresponding author. The data are not publicly available due to privacy restrictions.

## Supplementary Materials

The following supporting information can be downloaded at: https://www.mdpi.com/article/10.3390/nu15051156/s1, Figure S1: Prediction performance of individual and complete genetic risk score models, Figure S2: Accuracy and distribution of genetic risk scores in training and testing datasets, Figure S3: AUC-ROC in training and testing datasets.
