# Supplementary material for: Update of a Genetic Risk Score Predictive of the Plasma Triglyceride Response to an Omega-3 Fatty Acid Supplementation in the FAS Study
Source: Nutrients. 2023 Feb 25;15(5):1156. doi: 10.3390/nu15051156 (PMC10005670; doi:10.3390/nu15051156)
Supplement: Supplementary file 1 [file nutrients-15-01156-s001.zip › nutrients-2214116-supplementary.pdf]

Supplementary Material

Refinement of a genetic risk score predictive of the plasma triglyceride response to an omega-3 fatty acid supplementation in the FAS Study

Ellie Gauthier[1,2], Juan de Toro-Martín[1,2], Bastien Vallée-Marcotte[2], Simone Lemieux[1,2], Iwona Rudkowska[3], Patrick Couture[3], Marie-Claude Vohl[1,2*]

[1] School of Nutrition, Université Laval, 2440 Hochelaga Blvd, Quebec City, Quebec, Canada, G1V 0A6.

[2] Centre Nutrition, santé et société (NUTRISS)-Institut sur la nutrition et les aliments fonctionnels (INAF), Université Laval, 2440 Hochelaga Blvd, Quebec City, Quebec, Canada, G1V 0A6.

[3] Endocrinology and Nephrology Unit, CHU de Québec-Université Laval Research Center and Département de kinésiologie, Université Laval, Québec, QC, Canada, G1V 0A6.

* Correspondence: marie-claude.vohl@fsaa.ulaval.ca; Tel.: 418 656-2131 (4676)

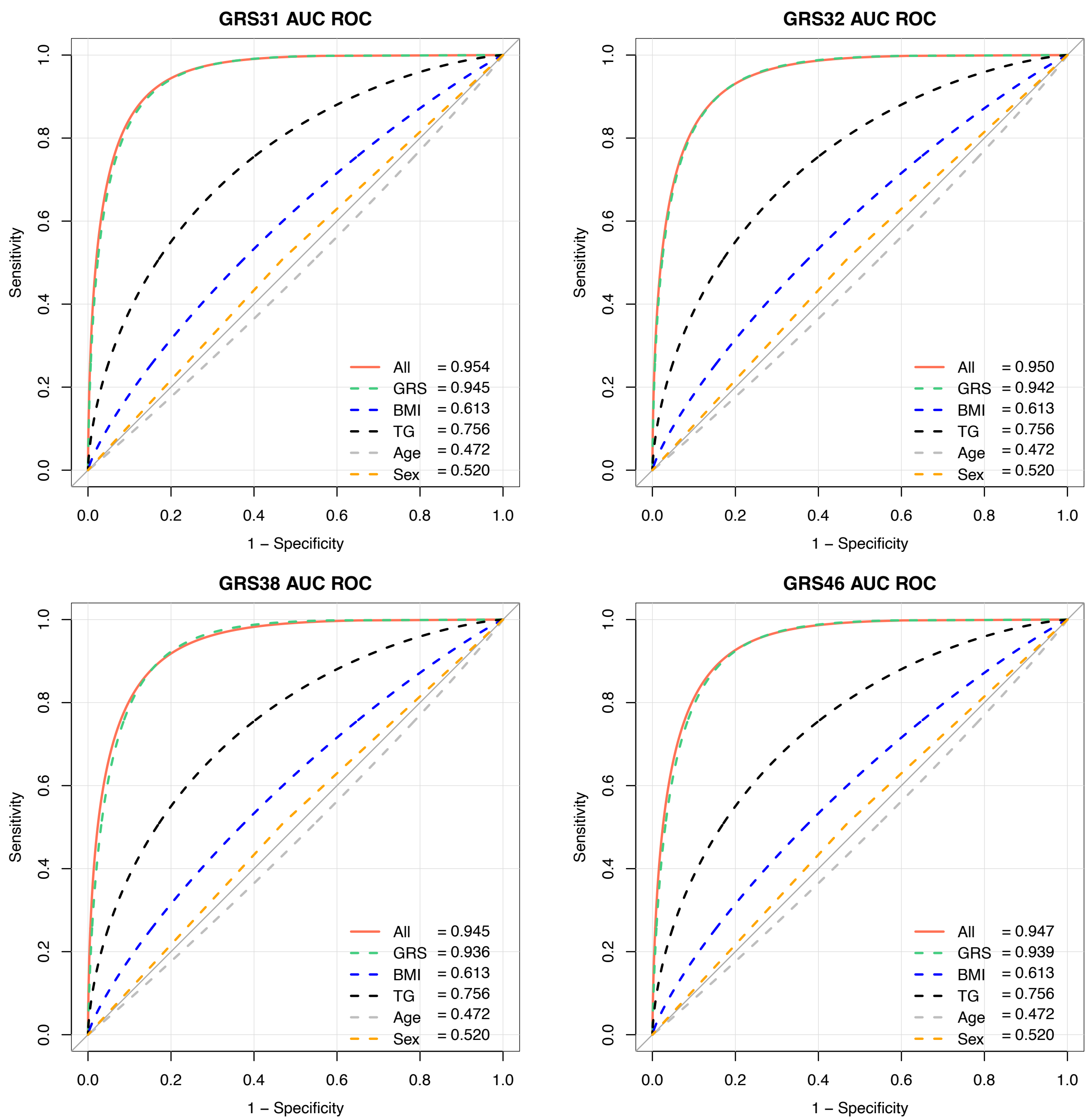

Figure S1. Prediction performance of individual and complete genetic risk score models. From left to right and from up to bottom are shown the receiver operating characteristic (ROC) curves of the final prediction models for GRS31, GRS32, GRS38 and GRS46 in the study population. ROC curves for each individual parameter included in the final models (age, sex, body mass index – BMI –, baseline triglycerides – TG –, genetic risk score – GRS–) are also shown on each panel.

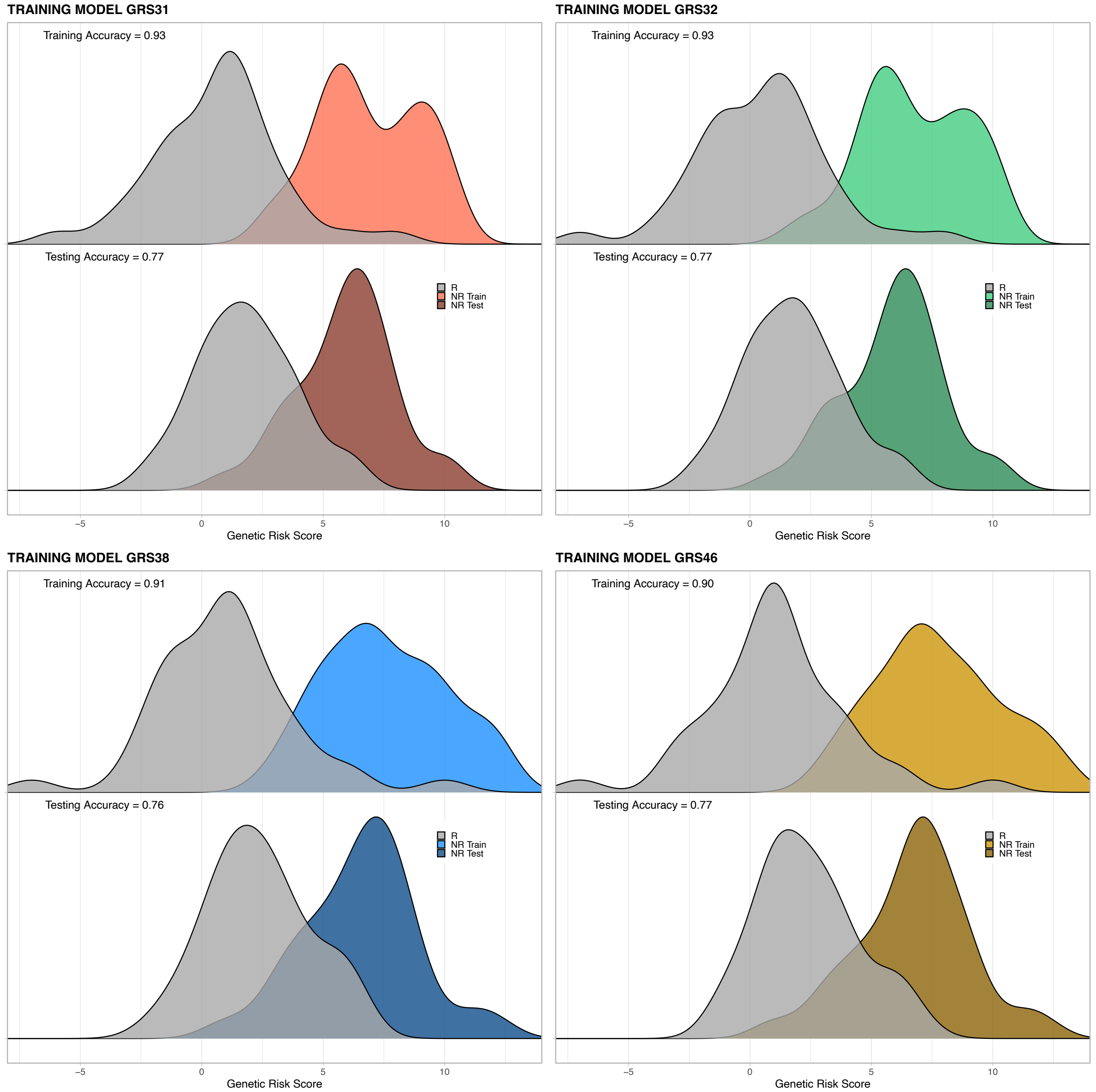

Figure S2. Accuracy and distribution of genetic risk scores in training and testing datasets. From left to right and from up to bottom are shown the density plots for the distribution of GRS31, GRS32, GRS38 and GRS46 in training and testing datasets. GRS: genetic risk score. R: responders, NR: non-responders.

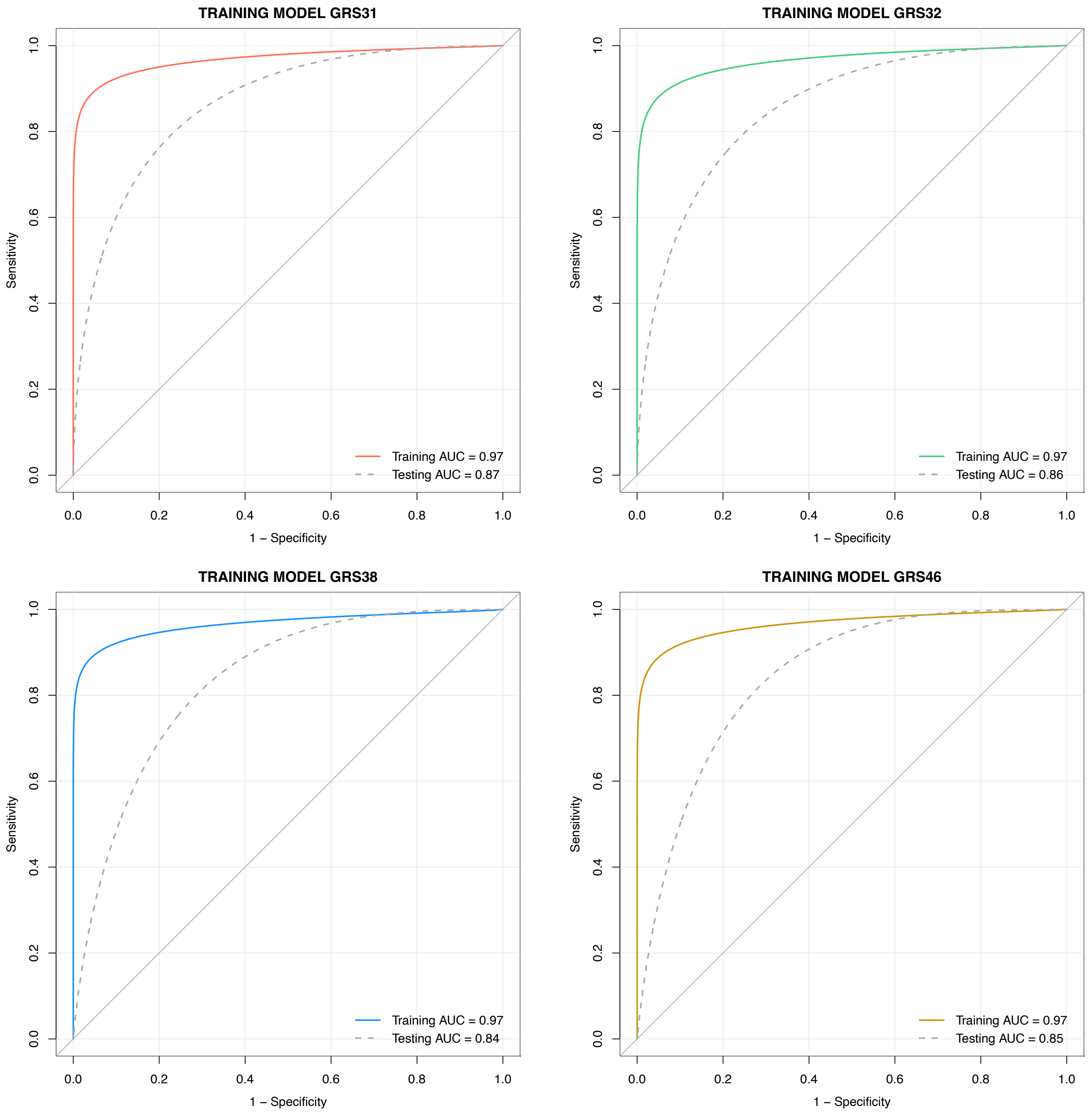

Figure S3. AUC-ROC in training and testing datasets. From left to right and from up to bottom are shown the receiver operating characteristic (ROC) curves of the final prediction models for GRS31, GRS32, GRS38 and GRS46 in in training and testing datasets.
